# Situating mobile health: a qualitative study of mHealth expectations in the rural health district of Nouna, Burkina Faso

**DOI:** 10.1186/s12961-017-0211-y

**Published:** 2017-07-12

**Authors:** Vincent Duclos, Maurice Yé, Kagoné Moubassira, Hamidou Sanou, N. Hélène Sawadogo, Gilles Bibeau, Ali Sié

**Affiliations:** 1 Department of Global Studies and Modern Languages, Center for Science, Technology & Society, Drexel University, 3101 Market Street, 2nd Floor Suite, Philadelphia, PA 19104 USA; 2 Nouna Health Research Center, Nouna, P.O. BOX 02, Burkina Faso; 3 Département d’anthropologie, Université de Montréal, Pavillon Lionel-Groulx, 3150 rue Jean-Brillant, Montréal, QC H3T 1N8 Canada

**Keywords:** Mobile health (mHealth), Expectations, Burkina Faso, Situated knowledge, Maternal health, Global health

## Abstract

**Background:**

The implementation of mobile health (mHealth) projects in low- and middle-income countries raises high and well-documented expectations among development agencies, policymakers and researchers. By contrast, the expectations of direct and indirect mHealth users are not often examined. In preparation for a proposed intervention in the Nouna Health District, in rural Burkina Faso, this study investigates the expected benefits, challenges and limitations associated with mHealth, approaching these expectations as a form of situated knowledge, inseparable from local conditions, practices and experiences.

**Methods:**

The study was conducted within the Nouna Health District. We used a qualitative approach, and conducted individual semi-structured interviews and group interviews (n = 10). Participants included healthcare workers (n = 19), godmothers (n = 24), pregnant women (n = 19), women with children aged 12–24 months (n = 33), and women of childbearing age (n = 92). Thematic and content qualitative analyses were conducted.

**Results:**

Participants expect mHealth to help retrieve patients lost to follow-up, improve maternal care monitoring, and build stronger relationships between pregnant women and primary health centres. Expected benefits are not reducible to a technological realisation (sending messages), but rather point towards a wider network of support. mHealth implementation is expected to present considerable challenges, including technological barriers, organisational challenges, gender issues, confidentiality concerns and unplanned aftereffects. mHealth is also expected to come with intrinsic limitations, to be found as obstacles to maternal care access with which pregnant women are confronted and on which mHealth is not expected to have any significant impact.

**Conclusions:**

mHealth expectations appear as situated knowledges, inseparable from local health-related experiences, practices and constraints. This problematises universalistic approaches to mHealth knowledge, while nevertheless hinting at concrete, expected benefits. Findings from this study will help guide the design and implementation of mHealth initiatives, thus optimising their chances for success.

## Background

Under the impulsion of governmental agencies, international institutions, non-governmental organisations and private sector players, mHealth is rapidly gaining importance on the global health agenda [1–5]. Belonging to a larger category of electronic health (eHealth) technologies, mobile health (mHealth) may be defined as medical and public health practice supported by mobile devices, such as phones, patient monitoring devices and other wireless devices [1]. The expansion of mHealth has been particularly rapid in sub-Saharan Africa in the context of increased access to mobile phones [6, 7]. In mid-2015, there were 386 million unique mobile subscribers in sub-Saharan Africa, and by 2020 it is expected that just under half (49%) of the population will have subscribed to a mobile service [8]. Under these circumstances, mHealth initiatives in sub-Saharan Africa have been taking many different forms [9]. The most common applications include the use of mobile phones to improve patient follow-up and medication adherence [10, 11], to support health workers in remote areas [12–14], or for data collection for public health and disease surveillance [15].

mHealth interventions in sub-Saharan Africa, and in low- and middle-income countries (LMICs) in general, are raising high expectations among development agencies, policymakers and researchers [16–18]. Individual benefits often associated with mHealth include an increased access to healthcare and health information, increased cost-efficiency of service delivery, improved ability to diagnose, treat and track diseases, and timely public health information [19]. A priority area of mHealth interventions in sub-Saharan Africa and in LMICs in general has been maternal health [20]. mHealth projects have, for instance, been implemented to strengthen pregnancy [21] and postpartum monitoring [22], or to improve skilled attendance at delivery [23]. mHealth has been widely expected to contribute in achieving the targets of the Millennium Development Goals for child and maternal health [24–26]. For instance, it has been suggested that mobile communication technologies hold the potential to reduce maternal health disparities related to cost, distance and inadequate health infrastructure [27].

Despite such expectations, it has become common within scholarly literature to lament the gap between expected and actual benefits and the lack of an evidence base regarding the actual impact of mHealth initiatives [3, 6, 28]. Evidence is dispersed, incomplete and unconvincing when time comes to assess the sustainable, cost-effective impact of mobile technology on health indicators. A primary concern has been the lack of evidence of successful scaling up, given that the vast majority of mHealth pilots implemented in LMICs have failed to translate or scale into health systems [29, 30]. This has brought research and implementing agencies to call for evidence meant at better informing investments within the sector [19, 31]. This situation also applies to the expectations and concrete impact of mHealth on maternal health in sub-Saharan Africa [20].

The production of evidence-based, universal knowledge about mHealth has so far paid scant attention to local expectations of direct and indirect users. Often, local perceptions and experiences tend to be reduced to issues of user acceptance and feasibility, as part of a broader concern with local barriers to the adoption of interventions [12, 32, 33]. This is problematic in many regards. To start with, the relationship between technology acceptance and successful mHealth project implementation is not straightforward [34]. Positive responses to mHealth do not necessarily lead to high uptake, as receptiveness is inseparable from various conditions, including socioeconomic factors, geographic barriers and quality of care [6, 23]. More importantly, to reduce local mHealth expectations to issues of acceptance and feasibility is to neglect the complexity inherent to both health-seeking behaviours [35–37] and processes of technology adoption [38–40]. This may sometimes border on technological determinism, with local contexts portrayed as blank slates upon which mHealth should be introduced without any great difficulty [41]. As a result, mHealth research runs the risk of developing and transposing knowledges which remain “*largely unconnected to the ways local communities identify, explain, and manage health problems*” [42]. This gap, or disconnection, may in turn undermine the influence of local knowledge on mHealth policymaking and project design. This paper may be read as a contribution aimed at bridging this gap from the ground up by focusing on mHealth expectations as situated knowledge, inseparable from specific experiences, practices and living conditions.

### MOS@N: mHealth in Nouna

This paper examines how mHealth is expected to affect access to maternal care in the Nouna Health District (NHD) in Burkina Faso. Data presented in the paper were collected as part of a wider mHealth research project currently being implemented in the NHD – the MOS@N initiative. Supported by the International Development Research Centre (IDRC/Canada), MOS@N (*mobile santé Nouna*) is being implemented by the Nouna Health Research Centre (Centre de Recherche en Santé de Nouna, CRSN), a national research centre located in rural Burkina Faso [43, 44]. This broader project aims to study the impact of mobile technologies on the utilisation of healthcare services by pregnant women and people living with HIV/AIDS. In this paper, we focus on maternal health.

Despite significant improvements [45, 46], high maternal mortality rates (341 maternal deaths per 100,000 live births) remain a major public health challenge in Burkina Faso. This is particularly the case in rural areas, in which most of the country’s 17 million inhabitants live. Estimates compiled by the Ministry of Health suggest that, in 2013, the proportion of women attending at least two antenatal care (ANC) visits was 72% for the whole of Burkina Faso and 70% for the NHD specifically [47]. In 2010, the proportion of women delivering in a health facility in rural Burkina Faso was estimated at 61.5% [47]. In rural areas, obstacles to maternal care access remain numerous, and include social, geographical and economic barriers, shortages of skilled health staff, lack of information on sexual and reproductive health, the cost of medical treatment, and negative perceptions of quality of care [48–52].

Under such circumstances, MOS@N aims to examine the impact of mHealth on health-seeking behaviours of pregnant women and on the follow-up of pregnancy in the NHD. The broader objective of MOS@N is to understand how mobile health technology can lead to more equitable health systems, while also examining key challenges, limitations and unintended effects. The project consists, among other things, in sending medical appointment reminders and medical advice to godmothers, who are community relays in charge of following up with pregnant women in their villages. To do so, godmothers are provided with a mobile phone (to receive messages) and a bicycle (to travel and communicate messages). Bringing together local primary healthcare centres (PHCs), health workers, ICT technicians, godmothers and public health researchers, MOS@N is currently being implemented in 26 villages, served by five different PHCs (with five more serving as a control group).

In this paper, we present qualitative data collected during the first phase of the MOS@N initiative, namely at the beginning of its implementation. The aim of this first phase was to document expectations towards mHealth among local women and health workers. The research presented in this paper addresses the following questions:

- How do women and healthcare workers in Nouna expect mHealth to impact healthcare access?
- What are the anticipated challenges and limitations associated with mHealth?
- How do such expectations – benefits, challenges and limitations – relate to local health-related practices, experiences and constraints?

## Methods

### Research design

The study was conducted within the NHD in Burkina Faso. The NHD is located approximately 300 kilometres to the northwest of Ouagadougou, the capital of Burkina Faso. It is one of the six districts of Boucle du Mouhoun Health Region and covers the geographical area of the Kossi Province in the western part of the country. The NHD comprises the town of Nouna with a total population of 29,297 inhabitants and a rural area of approximately 331,020 inhabitants. The health infrastructure of the NHD consists of one District Hospital (DH) in Nouna and 43 PHCs, out of which 10 PHCs are included in MOS@N.

MOS@N is a 36-month project that includes both qualitative and quantitative components. In this paper, we focus on the qualitative component, and specifically on data collected at the beginning of the project. By focusing on this initial phase of data collection, we aim to examine mHealth expectations prior to the full implementation of MOS@N. To collect data, semi-structured interviews and group interviews were conducted. The research was designed and implemented by a team of researchers from the CRSN, McGill University and Université de Montréal. Ethical approval for the study was granted by the ethics committee of the Ministry of Health of Burkina Faso and by the Institutional Research Ethics Board of the CRSN.

### Sample and recruitment

Qualitative data presented in this paper was collected over 2 months, in May and June 2014. Mixed purposive sampling methods were used to select participants [53]. As is usually the case with qualitative research, the aim was not to obtain a representative sample of the various categories of participants, but to gather a substantial body of information from them [54]. Participants can be divided into five different groups, namely (1) health workers in PHCs of the NHD; (2) godmothers participating in the MOS@N project; (3) pregnant women; (4) women with children aged 12–24 months; and (5) women of childbearing age. Table 1 presents the distribution of participants.

**Table 1:** Distribution of participants

Participants	Number	Description
Healthcare workers	19	19 interviews in 10 primary healthcare centres (PHCs)
Godmothers (*marraine*s)	24	24 interviews in 23 participating villages
Women with children aged 12–24 months	33	33 interviews in 10 PHCs
Pregnant women	19	19 interviews in 19 PHCs
Women of childbearing age	92	92 women, in 10 group interviews
Total no. of participants	187	

Following a purposive, expert sampling method, every health worker (n = 19) in participating PHCs (n = 10) was interviewed. Health workers belong to two subgroups, namely head nurses (*infirmiers chef de poste*, or ICP; n = 8) and midwives (n = 11). ICPs supervise the daily medical operations of the PHC. All the ICPs working in the participating PHCs were male. The midwives oversee maternal health services at the level of the PHC. All the midwives working in the participating PHCs were female.

Data collection also involved individual and group interviews with women from the local population of the NHD. First, semi-structured interviews were conducted with every godmother recently selected to participate in MOS@N. At the time of the interviews, 48 godmothers living in 26 villages had just been selected. Following a purposive sampling method, half of them (n = 24) were interviewed during this first phase of qualitative data collection. Interviews with godmothers did not focus on their actual experiences of MOS@N, which was just starting, but rather on their overall perceptions and expectations. Secondly, semi-structured interviews were conducted with pregnant women (n = 20) enrolled in MOS@N. Participants were selected following a purposive, non-proportionate quota sampling method, with at least one respondent in every participating PHC, with an average of two per PHC. Thirdly, semi-structured interviews were also conducted with women with children aged 12–24 months (n = 33). Participants were selected following a purposive, non-proportionate quota sampling method in which the sole criteria for inclusion was attending any of the 10 participant PHCs. At least two women were interviewed in every PHC. Finally, women of childbearing age (n = 92) were recruited to participate in group interviews (n = 10). These participants were selected following a purposive, non-proportionate quota sampling method in which the main inclusion criteria was geographical, since there was one group interview (with an average of nine participants per PHC) in every participating PHC.

### Data collection

We conducted semi-structured interviews with health workers, godmothers, pregnant women and women with children aged 12–24 months. Interviews lasted on average 30 minutes. Pregnant women and women with children aged 12–24 months were approached when coming to the PHC, either for an ANC visit or for a consultation with one or more children. Health workers and godmothers were approached as part of their broader participation in MOS@N and were met at their local PHC. The interviews were conducted by trained researchers from the CRSN. Interviews with health workers were conducted in French and interviews with godmothers, pregnant women and women with children aged 12–24 months were conducted in Dioula. Interviews were digitally recorded and transcribed. Those conducted in Dioula were transcribed into French. Interviews followed a pre-established interview guide, addressing various topics related to mHealth, access to maternal healthcare and mobile phones in general. As is usual with qualitative, semi-structured interviews, the main aim was to ask open-ended questions, which leave room to unexpected answers and are particularly adapted to discussing sensitive, health-related topics [55]. Semi-structured interviews were chosen over unstructured interviews, since while they allow the interviewer to depart from the interview guide, they are better suited to address specific issues when the research already has a fairly clear focus [56]. They also provide more consistency when there is more than one researcher involved in data collection, as was the case here. Qualitative interviews aim at gathering descriptions of the life-world of the interviewee, while remaining open for ambiguities and changes [57].

Group interviews, which were conducted with women of childbearing age, lasted between 60 and 90 minutes. They were also conducted in Dioula, digitally recorded and transcribed into French. Group interviews are particularly useful as part of such a multi-method design to clarify, extend, qualify or challenge data collected through other semi-directed interviews [58]. Each group interview included between 8 and 10 respondents. Interviews were moderated by trained researchers from the CRSN, whose role was to lead the discussion and elicit participation from all members [59].

### Data analysis

Data analysis followed common qualitative data analysis guidelines. The first analytic step taken was data organisation and indexing. Recorded interviews were transcribed into French, and read repeatedly by three of the authors, while noting down initial ideas. We then used content and thematic analysis methods [60]. First, two of the authors proceeded to content analysis, by doing an in vivo/emergent, open coding of the relevant data. ATLAS.ti qualitative data software was used for coding. This allowed the researchers to create categories, to group codes under higher order headings and to formulate a general description of the research topic [61]. Then, another author organised data into thematic categories, first by searching for themes, and then reviewing, defining and naming them [62]. Compelling extract examples were selected, analysed and related back to the research questions and literature. The authors then compared thematic analysis and content analysis, moving on to more focused coding, with particular emphasis on concepts related to mHealth expectations. As usual with qualitative analysis, the goal was not to achieve representativeness, but rather to identify meaningful patterns and variations [63].

## Results

Our main findings are presented in four sections. The first section introduces the general perceptions and common usages of mobile phones in the NHD, while focusing on health-related usages. The second section focuses on expectations towards mHealth and the MOS@N project, both among the population and health workers. The third section discusses challenges that participants expect the implementation of MOS@N, and mHealth in general, to be confronted with. The fourth section introduces limitations intrinsic to mHealth by examining the barriers to maternal care access that mobile phones are not expected to overcome.

### Mobile phones in Nouna

The level of experience with mobile phones among the population in Nouna is variable. Participants all know what mobile phones are since they have generally been present in villages for a few years, and phones are generally perceived very positively. The main purpose for which participants use them is to stay in touch with relatives living in other villages or parts of the country. Phones are also used to play music.

Women in the NHD also use mobile phones for health-related reasons. The most cited health-related usage is to communicate with a family member about an illness. Women with a mobile phone and traveling alone to the PHC may call home to update their relatives about their condition or that of a child. Phones are also used to ask their husband when he is away to take them to the PHC if they are not feeling well during pregnancy. Others like to call the PHC to know if there is any health worker present before traveling. Overall, mobile phones are perceived by women as a time-saving device, which reduces the need to travel in difficult conditions:

“*The mobile phone is a good thing because it reduces distances, saves us from bicycle breakdowns, since you can remain where you are and receive the information you need.*” (Woman of childbearing age, Biron Marka)

Mobile phones, however, are not readily accessible for many women in the NHD. Most participants mentioned having little experience using them, and the majority never owned one. Among the godmothers (n = 48) selected to participate in MOS@N, only one third (n = 16) previously had a phone to themselves, and half of those who possessed one had it for less than a year. In many cases, husbands or other members of the family have a phone, but women are sometimes given very little opportunity to use it. Women who do have a phone may also encounter obstacles using them. Purchasing telephone units may prove expensive, or their husbands may not allow them to use the phone to make calls. Two women explain:

“*I have bought a phone last year, and I had put a SIM card into it but my husband took it out and keeps it with him because he does not trust me. So right now I am listening to music with the phone.*” (Woman of childbearing age, Sobon)

“*We can use them to make calls but the problem is that here our husbands do not accept that we put SIM cards into the phones, so we can only use them for music.*” (Woman of childbearing age, Sobon)

These obstacles were reported by many of the women interviewed when discussing the presence of mobile phones in the NHD.

By contrast to local women, every health worker participating in this study already had a mobile phone for some time. Health workers have significant experience using phones, either for personal or work-related purposes. Every PHC comes equipped with a mobile phone as well as with prepaid telephone units. Although health workers do not refer to this as mHealth, phones have been playing an important role in the day-to-day activities of PHCs for a few years. Perhaps the most frequent usage of phones in the PHCs is to communicate with Nouna and with the DH. Health workers use them to transfer epidemiological data to the district level on a weekly basis as part of a national disease surveillance scheme. Health workers also use them to call an ambulance to transfer patients to the DH when they cannot treat them at the PHC. Medical references are also made over the phone. Furthermore, when the PHC runs out of a medicine, phones are used to call the pharmacy in Nouna to inquire about its availability. Health workers also sometimes call patients who have a mobile phone to follow up on their condition or that of a family member. These calls can occasionally replace a follow-up appointment. Overall, health workers consider mobile phones to be facilitators in the circulation of health information between physically isolated PHCs, colleagues in Nouna and, to a certain extent, patients and their families.

### Expected benefits: what mHealth can do

Pregnant women consulting at a PHC are all provided with a health booklet in which health workers note down their upcoming ANC visits in order to facilitate medical follow-up. However, most women in the NHD are illiterate and struggle to read ANC dates inscribed in the booklet. As an ICP noted, speaking of missed appointments:

“*Often, it is not because of ill will, but rather because of illiteracy and forgetting*” (ICP, PHC Labarani)

Many strategies are developed by pregnant women and health workers to deal with this situation. Often, women ask a literate member of the household or of the village, for instance, a schooled child, to read the date for them:

“*The date is written in the booklet. So you have to memorize it. To do so, once I come home I tell the date to my husband but also, since it is written, later I show it to my son who can read. This way it allows me to keep track.*” (Pregnant woman)

Another strategy used by health workers is to tell pregnant women to come back for their next ANC visit whenever there have taken all the daily iron supplements (30 doses) prescribed to them. When these strategies do not work, health workers will sometimes try to reach women who have missed their ANC via other members of the community, starting with community health promoters (*agents de santé communautaire*, or ASCs). ASCs are volunteers present in every village, playing an important role in informing the local population about health-related topics and events. Sometimes, health workers will call ASCs to ask them to inform certain patients about missed ANC visits:

“*For patients lost to follow-up, we make a list of women who have missed their antenatal consultations and we give it to the community health promoter. He will then inform these women.*” (Midwife)

In some villages ‘emergency units’ (*cellules d’urgence*) have also been put in place by ASCs to manage obstetrical emergencies. Health workers sometimes ask these units to identify pregnant women that do not attend ANC visits and refer them to the PHC.

Despite such solutions, ANC consultations are too often missed. Health workers participating in our study believe that mHealth, and MOS@N in particular, can improve the monitoring of patients and reduce the numbers of patients lost to follow-up. Computers provided to the PHCs as part of MOS@N are expected to play an important role in this regard. A critical component of the project, as is often the case with mHealth, is the electronic health records (EHR) system in which health workers register patients when they come for their first ANC visit. The EHR allows health workers to keep track of their patients’ ANC appointments:

“*I think it fits very well because once we register pregnant women into the computer it will be useful to identify patients lost to follow-up. The computer will alert us about patients that miss appointments so that we can look for them.*” (Midwife)

Furthermore, the EHR automatically generates voice message reminders of upcoming ANC consultations. These messages are received by a godmother to whom certain patients are assigned. Local health workers thus expect mHealth to replace and improve the former, more informal communication channels via the ASCs. Instead of establishing, by using a mix of paper medical records and memory, a list of patients then communicated to the ASC, health workers can now let the EHR automatically generate the list and communicate reminders to godmothers, whose role is also more defined than that of ASCs.

Participants almost unanimously welcome godmothers and the role they are expected to play:

“*I think they have an important role to play, helping us to remember the date of our consultation.*” (Démé Salimata, pregnant woman, PHC Dara)

Women participants, however, believe that the role of godmothers should not be restricted to transmitting the information received via mobile phones. They suggest that mHealth should be conceived as a core component of a network aimed at both raising awareness about maternal health and assisting them during pregnancy. For instance, some women have mentioned that godmothers could get closer to health workers to learn things and spread that knowledge within communities. They expect mHealth to bring about a network of support that would complement existing resources. Godmothers themselves agree on the fact that their role is not restricted to conveying information about upcoming ANC appointments. A godmother summarises this overall perception in this way:

“*Our role is to offer guidance so that women go to the PHC and also to provide information to the population. … I have accepted this role because we act as intermediaries between health workers and our communities.*” (Godmother)

This notion of being an ‘intermediary’ is largely shared by health workers. Two ICPs explain:

“*Since godmothers live within the communities, communities will feel more concerned by our activities and they will understand even better that the PHC belongs to them.*” (ICP)

“*They are extremely useful because here at the PHC level, we do not know people in the community. Because they live in the villages, they can become our mouths and our ears with the population. … I’m a stranger here, but they know everyone. Who else could get them to participate to our activities?*” (ICP)

In other words, all participants conceive of the mobile phone as a medium that contributes in establishing new relationships with the PHCs. mHealth is expected to transform local health-seeking practices by enabling a network of support at the village level, and by building community ties.

### Expected challenges: what mHealth must be careful doing

Participants expect mHealth implementation in the NHD to face several challenges, which can be divided into five types: (1) technological barriers; (2) organisational challenges; (3) gender issues; (4) confidentiality concerns; and (5) unplanned aftereffects.

#### Technological barriers

Participants expect that implementing mHealth solutions in the NHD will involve overcoming important technological barriers. Many women have noted that mobile network availability and stability in their village could be a major obstacle in the day-to-day work of the godmothers. In some villages, it is not uncommon to spend a few days without network connectivity. The absence of electricity was also mentioned as a challenge, since it implies that mHealth projects need to include a reliable energy source. MOS@N, for example, provides godmothers with solar panel mobile chargers. However, even with such a solution, the lack of power supply can still be a problem when participants need to travel, or to sleep away from home. A godmother explains:

“*The problem that we have is that at the moment we sleep in the bush, to work in the fields. So when the battery will discharge we won’t be able to travel by night to plug it to the panel.*” (Godmother)

Technological challenges also apply to PHCs, the most important one being the lack of experience of health workers with computers:

“*The phone is easy to use, but computers, when you never had one, we will need some training because otherwise it won’t be easy for us to use it.*” (Midwife)

#### Organisational challenges

Participants also expect that mHealth presents organisational challenges, the most important of which is a possible increase in the workload of the health workers. With few exceptions, health workers anticipate that a project like MOS@N, for instance, will increase their workload despite its automation of certain tasks.

“*It will increase our workload. Because if we introduce computers here, we will still have to do everything we did before on paper, before typing it on the computer. At the beginning, in any case, it will be an extra burden.*” (Midwife)

Health workers consider that this increase in their workload could eventually compromise the successful implementation of mHealth, given that lack of time is already an issue in their daily practice. Expected organisational challenges also include providing quality training to project participants, rigorous monitoring of the mHealth activities by the implementing team, well-defined tasks, and measures to sustain the motivation of participants.

#### Gender issues

Local women, including godmothers, have also mentioned that gender issues could compromise successful mHealth implementation. As mentioned earlier, some husbands will not allow them to own and use a phone to make calls. Women thus expect that some godmothers will not be able to fulfil their duties because of marital pressure:

“*Here godmothers have just been selected but they won’t be able to do their work since their husbands won’t let them.*” (Woman of childbearing age, Bagala)

Godmothers themselves have mentioned that their mHealth duties could be a source of conflict within the household. Others believe that it might eventually be their husband who will be in charge of the phone to be provided as part of MOS@N.

#### Confidentiality concerns

The idea that phones receiving confidential health information could be controlled by the husbands of godmothers obviously comes with all sorts of ethical concerns. However, even beyond this scenario, many have expressed concerns about the confidentiality of health information circulating on the network:

“*I do not really know, but those who know think it is positive. But I know that it is not only positive. Can’t it also be a source of conflict? Phones can reveal secrets and then it is not good.*” (Godmother)

Confidentiality can be particularly problematic given that pregnancy is often kept secret in rural Burkina Faso, especially when young women are involved. A few health workers expressed concerns about the discretion of godmothers in this regard. Others mentioned that confidentiality could be compromised if information sent over the network was intercepted by local network providers.

#### Unplanned aftereffects

A few health workers mentioned that mHealth could produce unplanned aftereffects in the long run. It has been suggested that, with the implementation of MOS@N, women will now expect some sort of medical follow-up from the godmothers, who will in turn expect to receive automated reminders. mHealth could eventually disrupt existing practices and lead to an overreliance on the network:

“*The downside is that when women will get used to this system, they won’t come if we don’t call them. They will wait until we call before coming. So if the project is not there in the long term we will experience problems with our patients.*” (Midwife)

In sum, mHealth could have negative long-term consequences on the health-seeking behaviour of pregnant women if it is not adopted permanently as part of the local health system.

### Intrinsic limitations: what mHealth cannot do

When asked about the main barriers to maternal care access in the district, participants generally do not consider poor communication to be one. For instance, according to health workers, most pregnant women in the NHD are already aware of the importance of attending ANC consultations and therefore aim to keep their appointments. However, pregnant women face many obstacles in trying to do so, and there is little that mHealth is expected to do in this regard. These obstacles can be divided into three main categories, namely geographical accessibility, economic factors and gender inequalities.

For participants, the single most important obstacle to maternal care in the NHD is poor geographical access to PHCs. Considering that they often live between 5 and 10 kilometres from the nearest PHC, travel can become complicated for pregnant women. This is especially the case during the rainy season, when roads become impassable, being sandy, clayey, if not literally flooded for weeks. Most women walk or cycle to the PHC, but as they get closer to the due date they will sometimes use a motorcycle, and therefore, when roads become impassable, motorcycles or bicycles are no longer an option. Walking can become perilous too. Some villages are then literally isolated from their PHC. Under such circumstances, health workers are clear that the rainy season is the most important factor affecting ANC attendance as well as the number of home births:

“*Even if we try to raise awareness, during the rainy season women don’t come because of poor road conditions. Even if ask the ASC or a godmother to go and see them, they won’t come. During the rainy season, there are many home births.*” (Midwife)

“*Women in labour coming from Kongoba or Karasso may deliver on their way here. They may also turn back and return home. During the rainy season many also do not show up for their antenatal or postnatal consultations.*” (Midwife)

A few participants suggest that mHealth can contribute in hampering the impact of such isolation:

“*The introduction of mobile phones in the health care system will be a very good thing because our village is surrounded by water so there are moments during the rainy season when we become isolated and women can’t go to the PHC for their antenatal consultations, so using the phone we could communicate and find solutions in the cases.*” (Godmother)

In general, however, participants do not consider mHealth to be a particularly adequate solution to poor geographical access. What they ask for is an increase in the number of PHCs. Otherwise, geographical access is expected to limit the positive impact of mHealth in the NHD.

Another obstacle to maternal care access is economic hardship, which can take many forms. Although ANC consultations are provided free of charge and delivery is relatively affordable (900 CFA francs or 1.50 USD), medication and hospitalisation can represent big expenses for households. Transfers to the DH in Nouna represent an even more important burden. Some explained that, when becoming indebted with the PHC, for instance, for expenses not related to maternal care, they may avoid coming back until they can pay. Another economic obstacle comes from the livelihood of households in rural Nouna. A midwife explains:

“*Pregnant women are regularly coming to the PHC during the dry season but during the rainy season they may still come but not keep with their appointments. The reason is women need to work in the fields so it makes things more complicated.*” (Midwife)

Again, the potential impact of mHealth on such economic factors is intrinsically limited.

Gender inequalities were also mentioned by participants as a barrier to maternal care access. In most households, health-related decisions are primarily taken by men:

“*When we are pregnant it is our husband who makes decisions relating to pregnancy. We share some responsibility but the final say rests with the husband, who can take us to the PHC if we are sick.*” (Woman of childbearing age, Toni)

Most women mentioned that their capacity to pay for healthcare was dependent on their husbands’ will. While most husbands apparently think that maternal care is important, others do not take good care of their wives or do not encourage them to visit the PHC:

“*It is for their own health that women will go to the PHC for their antenatal consultation. If she has a bicycle she can use it, otherwise she will walk. Husbands here do not tell their wives to go to the PHC for their consultation.*” (Woman of childbearing age, Sobon)

Another woman explains that their husbands might refuse to pay for medication, or insist that they should be working instead of going to the PHC:

“*If you have money you can pay your own medication but if you don’t then you come back home with the prescription which you will give to your husband so that he can pay for it. Then, your husband will tell you that you never had permission to go to the PHC in the first place. For this reason, many women do not want to go to the PHC.*” (Woman of childbearing age, Sobon)

Gender inequalities thus contribute in keeping some pregnant women away from the PHC. While the impact of mHealth on gender-based decision-making within the household remains to be examined, this is not an expectation that participants have widely associated with mHealth. As mentioned earlier, gender inequalities are, by contrast, sometimes expected to be a factor impending the successful implementation of mHealth in the NHD.

## Discussion

Our findings suggest that expectations associated with mHealth in the NHD should be considered as situated knowledge that complicates universal mHealth knowledge or evidence building [64, 65]. As mentioned earlier, beyond the usual focus on acceptability and feasibility, research examining local expectations of mHealth in sub-Saharan African is, to our knowledge, virtually non-existent. The results of this study, however, do not come as a surprise. For some time now, social science research has employed qualitative methods to show that knowledge about health and illness is always situated in particular times and places [66–68]. To speak of knowledge as situated is to insist on the fact that it represents the understanding that local actors give of a particular phenomenon built in a community of practice [69]. This applies to mHealth expectations, of benefits, challenges and limitations, in the NHD.

Participants in the study generally expect mHealth to produce positive, tangible benefits. Health workers believe that mHealth will improve patient monitoring, by helping them identify pregnant women who missed ANC visits. They also insist on the role of computers, and of the EHR, in automatically generating appointment reminders and improving patient monitoring. Importantly, however, participants do not conceive of mHealth, and MOS@N in particular, as an essentially technological intervention. Put differently, the key expected contribution of mHealth is not a given number of messages sent and received over a technological network, but rather the establishment of a network of support which goes along with existing healthcare-seeking behaviours and constraints. In this regard, one of the pressing local needs which participants associate with mHealth is to build stronger relationships between the PHCs and communities. This is particularly the case of MOS@N since it involves godmothers, who are community relays entirely dedicated to maternal healthcare. Mobile phones are thus not expected to be mere instruments conveying information, but rather to assist godmothers in the mediation of relationships between PHCs and communities. It is this work of mediation, which is expected to create a network, and not the other way around, that would positively influence health-seeking behaviours of pregnant women living in the NHD. To suggest that these expected benefits are situated is to underline the fact that they represent collective, partial, dynamic and pragmatic knowledge, shaped by the situations and networks in which people participate [70].

The same applies to expected challenges. Whether they be technological, organisational, gender related or involving confidentiality, expected challenges are to be understood in relation to experiences (inexperience with technology, etc.), practices (workload, sleeping over in the fields, etc.), daily constraints (lack of power and of network connectivity, etc.) and power relations (gender inequalities, etc.) that constitute the lifeworld of participants. Neglecting this may lead to a misinterpretation of the challenges at hand, and ultimately to poor project design and disappointing results [34, 40, 71]. To take the example of gender relations, a review of literature on the influence of mHealth interventions on gender relations in developing countries shows that, while projects can increase women’s autonomy in seeking health services, projects that are not carefully designed may also reinforce existing power imbalances.

The findings of this study warn against attempts to design mHealth projects based on expectations that would be disconnected from the specificity of the sites where they aim to be implemented, and of the situations they aim to improve. This is all the more important for mHealth projects aimed at modifying local healthcare-seeking strategies, like MOS@N. As social science scholarship has aptly shown, healthcare-seeking strategies are influenced by many factors that include the perception and experience of illness, seasonal variations, past experiences with healthcare services, family support and socioeconomic status [72–74]. Healthcare-seeking behaviours in rural Burkina Faso are obviously no exception [51, 75, 76]. A study has, for instance, shown how delay in decision-making to use skilled care during pregnancy in rural Burkina Faso was caused by many factors that include poverty and the lack of empowerment of women, given the secondary role they often play in the decision-making process [77]. Being consistent with such literature, our participants do not expect mHealth to modify health-seeking behaviours without addressing the underlying factors. To take the example of geographical accessibility, being aware of an upcoming ANC is sometimes not enough. Pregnant women may still have to walk to the PHC, often in difficult conditions. Additionally, many studies have shown how distance to health facility is a major determinant of maternal care utilisation, including likeliness to seek ANC and to deliver in a health facility, in rural Burkina Faso [51, 78, 79].

The expected limitations of participants are also consistent with a review of literature which suggests that mHealth holds great potential for improving the use and quality of maternal health services, provided that women are not restricted due to their position in society, lack of finance or means of transport [25]. For instance, studies have shown that, although mobile phones can improve linkages between pregnant women and their PHC provider, other barriers to care, such as geographical distances, poverty, quality of care and sociocultural factors, influence the receptiveness of the mobile phone interventions [23]. This is also consistent with wider mHealth research suggesting that the scope of mHealth for treatment compliance, including appointment reminders, is limited in areas where access to health services is poor or inconsistent [19]. mHealth can complement, but not replace, adequate healthcare access.

Regarding MOS@N, data collected at this early stage of the study allowed us to modify the project in ways that we thought better integrated local expectations about benefits, challenges and limitations. For the sake of brevity, we will mention three main domains in which these expectations were taken into consideration. First, data presented here convinced us to pay special attention to the relationship between mHealth and gender inequity, and while further implementing and monitoring MOS@N, we have carefully considered the potential adverse effects of the project in this regard. We have also conducted interviews, which were not initially planned, later in the study specifically on this topic, including with husbands of godmothers. These interviews are currently under analysis and should lead to more publications. Secondly, MOS@N has indeed encountered many of the technological challenges that participants in this study had anticipated. As a result, MOS@N’s technical infrastructure has been significantly altered since it was launched [44]. Some of its more advanced technological features were removed, while a great amount of fine-tuning and tinkering was necessary to keep the project afloat. Thirdly, following these initial interviews, the role of godmothers was transformed in important ways. While initially it primarily consisted in the reception and conveyance of messages, godmothers have gradually come to play a more important role in assisting pregnant women. For instance, most godmothers decided to accompany pregnant women to their appointments, and to assist midwives during deliveries. Taking data collected here into consideration, we have encouraged such initiatives, although they were initially not planned. Although we are still examining the impact of this transformation in the role of godmothers, the data presented convinced us that this enlarged role better fits the needs of the communities in which MOS@N is being implemented. This new role affects the entire MOS@N project, since mobile technology, albeit important, now appears as only one aspect of a much larger support network at the village level. As such, it is plausible that modifying MOS@N in this way allowed us to at least partially overcome some of the limitations of mHealth to improve health seeking behaviours.

### Limitations

This study presents limitations. First, the fact that data was collected as part of a mHealth initiative, MOS@N, may have influenced the expectations of participants about mHealth. This is likely the case for participants preparing to be active users of MOS@N (godmothers and health workers), or for those that might benefit from it (pregnant women). This study can therefore not claim to document mHealth expectations in the general population of the NHD, since many participants were selected because of their involvement in MOS@N. A second limitation comes from the fact that women participating in the study were recruited at local PHCs. This sampling procedure facilitated the recruitment of participants, allowing the collection of data before the full-fledged implementation of MOS@N. However, it left out women not using PHCs at all. It is plausible that these women have a different perception of mHealth initiatives aimed at encouraging them to attend maternal care at the PHCs. Further, recruiting women at the PHCs may have influenced their discourses about the PHCs themselves, for example, on the quality of care provided. The study is also possibly limited by the fact that most interviews were conducted by male interviewers, which may have influenced the discourses of women participants about gender relations, among other topics. Finally, since the interviews (except for health workers) were conducted in Dioula, it is possible that the meaning of certain local idioms, or discourses, may have been altered in the translation process.

## Conclusion

This study suggests that mHealth expectations should be considered as collective, partial and pragmatic knowledge shaped by the situations and networks in which people participate. mHealth expectations are not static, but rather dynamic expressions of the ways local communities identify, explain and manage health problems, which are in turn inseparable from specific experiences, practices and constraints. By insisting on the situatedness of mHealth expectations, this study complicates universalistic approaches to mHealth, which even when taking local context into consideration tend to assume an overriding rationality that determines universal goals and expectations. It calls in turn for careful project design and policymaking, attentive to the knowledge and practices of local communities. Ultimately, findings from this study may help guide the design and implementation of mHealth initiatives, thus optimising their chances for success.

## Abbreviations

ANC: antenatal care
ASC: community health promoters (“agent de santé communautaire”)
CRSN: Centre de Recherche en Santé de Nouna
DH: district hospital
EHR: electronic health record
ICP: head nurse (“infirmier chef de poste”)
LMIC: low- and middle-income country
NHD: Nouna Health District
PHC: primary health centre (Centre de Santé et de Promotion Sociale).
